# Nutraceutical and Ethnopharmacological Properties of *Vangueria infausta* subsp. *infausta*

**DOI:** 10.3390/molecules23051089

**Published:** 2018-05-04

**Authors:** Alfred Maroyi

**Affiliations:** Medicinal Plants and Economic Development (MPED) Research Centre, Department of Botany, University of Fort Hare, Private Bag X1314, Alice 5700, South Africa; amaroyi@ufh.ac.za; Tel.: +27-719-600-326

**Keywords:** ethnopharmacological, nutraceutical, rubiaceae, tropical Africa, *Vangueria infausta* subsp. *infausta*

## Abstract

*Vangueria infausta* subsp. *infausta* (VI) is a nutraceutical with plant parts valued in several cultures for its medicinal properties. Traditionally, VI is used against gastro-intestinal disorders, malaria, pneumonia, cough, menstrual problems, parasitic worms, chest complaints, snake bites, infertility, fever, candidiasis and abdominal pains. This study aims to critically summarize the nutraceutical properties, phytochemistry and pharmacology of VI with a view to provide baseline data required for further investigations on this plant. Relevant information on the nutraceutical and ethnopharmacological properties of VI was collected from established scientiﬁc databases such as ScienceDirect, SciFinder, PubMed, Google Scholar, Medline, and SCOPUS. Additionally, books, book chapters and conference papers were also consulted to access other important information. Comparative analysis of the literature revealed that VI is an important neutraceutical plant in east and southern Africa, used as herbal medicine in 69% of the countries where the species is native. Phytochemical studies revealed the presence in VI of fatty acids, flavonoids, iridoid lactones and triterpenoids. Based on *in vitro* and animal studies, the species exhibited antibacterial, antimycobacterial, antifungal, anti-inflammatory, antileishmanial, antioxidant, antiplasmodial, antifeedant and prostaglandin synthesis inhibitory activities. Pharmacological studies have provided supporting evidence for the therapeutic value of VI, however, detailed toxicological and clinical trials are required to assess efficacy of the species.

## 1. Introduction

**Figure 1:**
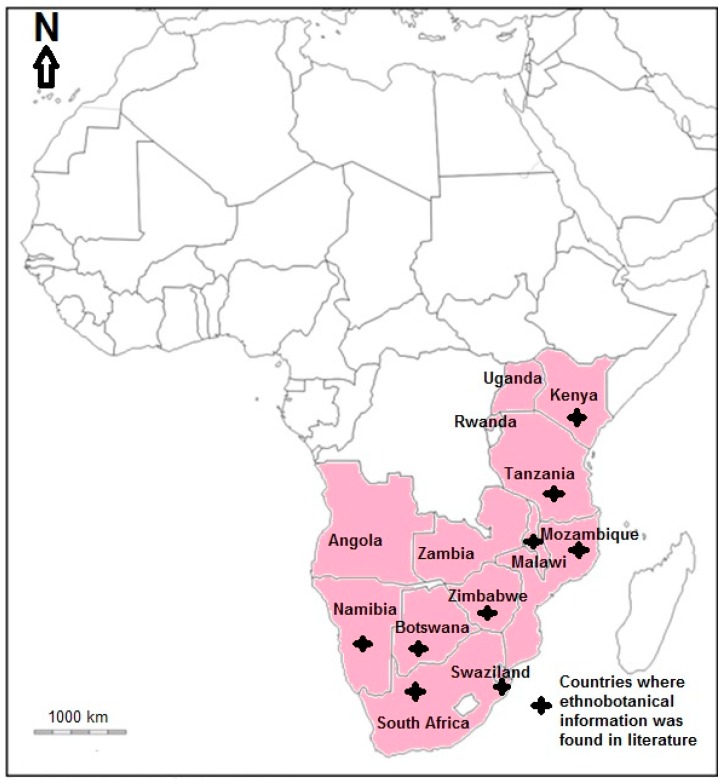
Distribution of *Vangueria infausta* subsp. *infausta* in the mainland tropical Africa.

*Vangueria infausta* Burch. subsp. *infausta* (VI) is native to Angola, Botswana, Kenya, Malawi, Mozambique, Namibia, Rwanda, South Africa, Swaziland, Tanzania, Uganda, Zambia and Zimbabwe [1,2,3] (Figure 1). *Vangueria infausta* subsp. *infausta* is a member of the Rubiaceae (coffee, madder or bedstraw) family. The Rubiaceae family is one of the five largest families of flowering plants in the world, with over 637 genera and 13,000 species [4]. Lantz and Bremer [5] analysed 180 species using nuclear ITS and the chloroplast markers trnT-F and rps16, and concluded that the genus *Vangueria* Juss. is monophyletic. *Vangueria infausta* subsp. *infausta* formed a strongly supported clade together with *V. esculenta* S. Moore, *V. madagascariensis* J. F. Gmel. and *V. proschii* Briq. characterized by narrowly oblong calyx lobes [5]. The genus *Vangueria* consists of over 50 species distributed in Africa south of the Sahara with one species (*V. madagascariensis*) occurring in Madagascar with the centre of diversity of the genus in east Africa, in Kenya and Tanzania [5]. The generic name *Vangueria* is believed to be derived from the native name of the type species *V. madagascariensis*, “voa vanguer”, a common name widely used for the species in Madagascar [6]. The species and subspecies name “*infausta*” is a Latin word which translates to disastrous or unlucky, probably referring to tribal superstition surrounding the species in southern Africa [6,7]. In South Africa, VI is believed to possess evil powers and its wood is not used for making fire as it is believed that it could cause cattle to bear only male offsprings [8]. *Vangueria infausta* is divided into two subspecies, *V. infausta* subsp. *infausta* and *V. infausta* Burch. subsp. *rotundata* (Robyns) Verdc. on the basis that subsp. *rotundata* is characterized by more extensively branched inflorescences, fully developed leaves at the time of flowering and more pointed corolla lobes [1,2]. *Vangueria infausta* subsp. *infausta* is known by various vernacular names in different countries throughout its geographical range (Table 1).

*Vangueria infausta* subsp. *infausta* is a deciduous shrub or small tree that grows up to 3 m to 8 m in height with a short multi-stemmed trunk supporting hanging branchlets [3]. The branchlets are covered with short, woolly hairs, and the bark is grayish to yellowish brown in colour, smooth and prone to peeling in irregular strips. The leaves are medium to large in size (5 to 10 mm long), thickly furry, opposite, light green in colour, net veined, covered with soft and velvety short hairs [1,2,3]. The leaf margins are entire, elliptic to ovate in shape, often twisted and rough to touch in older plants. The flowers are small and greenish white to yellowish in colour and are borne in small, multi-branched groups, densely clustered along short, lateral branches [1,2,3]. The fruits are round, glossy dark green when young and changing to a light brown colour when ripe. The fruit is usually borne singly or in pairs on twigs below the leaves [40]. It has been recorded in various types of woodland, wooded grassland, open grassland, scrub, often on rocky hillsides, kopjes, in sandy valleys, dry forest, near the sea on sand dunes [1,2,3].

The use of VI as a nutraceutical is mainly due to the nutritional and medicinal properties associated with the species. Fruits of VI are commonly sold by local street vendors in east and southern Africa, particularly in Botswana, Malawi, Mozambique, Tanzania, Zambia and Zimbabwe [10,14,23,41,42]. Roots and stems of VI are commonly sold as herbal medicines in Gauteng Province in South Africa [21,43]. Okole and Odhav [44] argued that the fruits of VI have potential for domestication in tropical Africa based on the widespread utilization of the fruits. Research by Van Wyk [22] revealed that the fresh or dried fruits of VI have potential in the commercial development of new food and beverage products such as jams and other processed products. Based on its importance as a source of edible fruits, VI has been introduced in trial plantings in the Negev desert in Israel [23]. Fruit of cultivated trees may be up to ten times larger than trees in the wild and between 2.5 and 5 kg of fruit per tree were attained in trial plantings in Malawi [23]. *Vangueria infausta* subsp. *infausta* is cultivated and/ or managed in home gardens as a food plant in Tanzania [41] and the Limpopo province in South Africa [45]. The species is an important food plant in Mozambique and Tanzania and it is considered a priority for conservation in these countries [13,46]. Therefore, this review is focused on VI, a species considered essential to local livelihoods in east and southern Africa as both a food plant and herbal medicine source. Therefore, this study was aimed at reviewing the nutraceutical and ethnopharmacological properties of VI.

## 2. Materials and Methods

**Figure 2:**
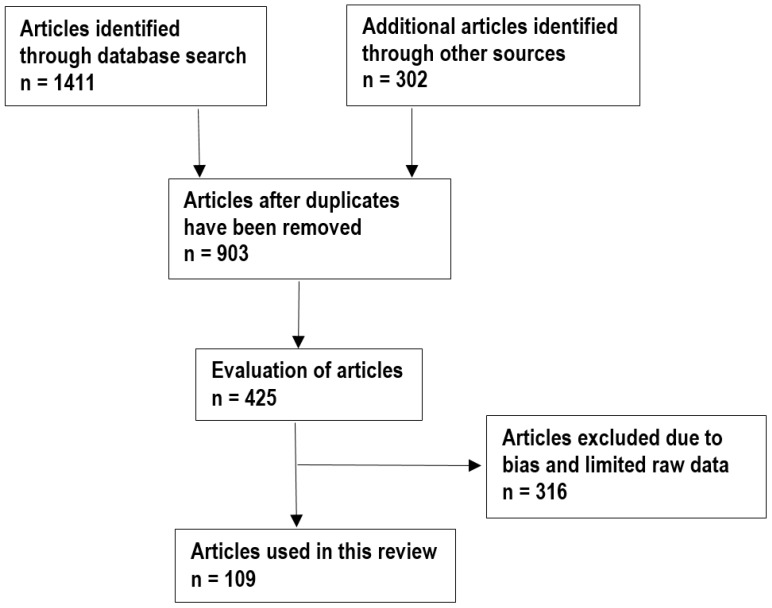
Flow diagram showing the literature search and selection processes.

A literature search for information relevant to the nutraceutical and ethnopharmacological properties of VI was carried out from October 2017 to February 2018. Information was obtained from the main online scientific literature sites, including ScienceDirect, SciFinder, PubMed, Google Scholar, Medline, and SCOPUS. Searches were also undertaken in the University of Fort Hare library, and dissertation search engines like ProQuest, Open-thesis, OATD and EThOS. The keywords used in the search included “*V. infausta* subsp. *infausta*”, “*V. infausta*”, the synonyms of the species “*V. barnimiana* Schweinf., *V. infausta* var. *virescens* Sond., *V. munjiro* S. Moore, *V. tomentosa* Hochst. and *V. velutina* Hook.”, English common names “African medlar, false medlar, large wild medlar, wild medlar and velvet wild medlar”. Additional searches were also carried out using the keywords “biological properties + VI”, “ethnobotany + VI”, “ethnomedicinal uses + VI”, “ethnopharmacological properties + VI”, “food uses or values + VI”, “indigenous knowledge + VI”, “livelihood needs + VI”, “local knowledge + VI”, “medicinal uses + VI”, “nutraceutical properties + VI”, “pharmacological properties + VI”, “phyochemistry + VI”, “traditional uses + VI” and “traditional ecological knowledge + VI”. The nature and number of keywords used in this study generated 1411, articles as shown in Figure 2. Only articles published in English language were considered for inclusion as translation facilities for other languages were not available. Articles that were included in this study focused on the medicinal uses of VI including details of the plant parts used and diseases treated, food value of VI including plant parts consumed and methods of preparation; phytochemical and pharmacological properties of VI. After removal of duplicates and application of the inclusion and exclusion principle, a total of 109 articles published between 1962 and 2018 were included in the review, published in international journals (76), books (13), dissertations and theses (11), scientific reports or working papers (four), websites (three) and book chapters (two).

## 3. Medicinal Uses

**Figure 3:**
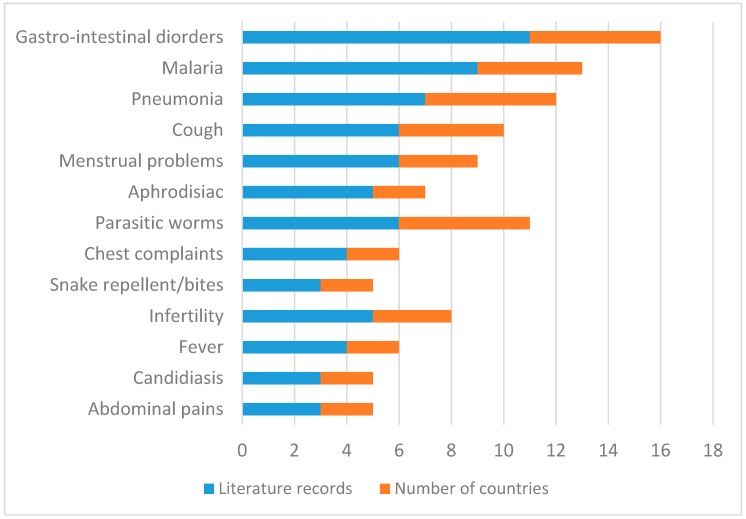
Diseases and ailments treated by *Vangueria infausta* subsp. *infausta* in east and southern Africa.

The bark, fruits, leaves, roots, seeds and shoots of VI are used to treat and manage 43 human diseases in east and southern Africa (Table 2). Ethnomedicinal information has been found in Botswana, Kenya, Malawi, Mozambique, Namibia, South Africa, Swaziland, Tanzania and Zimbabwe, representing 69.2% of the countries where VI is native (Figure 1). Gastro-intestinal disorders, malaria, pneumonia, cough, menstrual problems, aphrodisiac, parasitic worms, chest complaints, snake repellent or remedy for snake bites, infertility, fever, candidiasis and abdominal pains (Figure 3) are the most commonly treated human diseases and ailments using concoctions prepared from VI. In traditional medicine, root infusions of VI are used against abdominal pains in Kenya and Zimbabwe [37,39,47] and to slow down heartbeat in Botswana and South Africa [48,49]. Leaf and root decoction of VI is used as an aphrodisiac in South Africa and Tanzania [6,24,33,40,50], purgative in South Africa and Swaziland [28,31], remedy against diabetes in Tanzania [51] and fever in Nambia and Tanzania [52,53,54], cold and headache in Namibia [53,54], hernia in Tanzania [49], and malaria in Malawi, South Africa, Swaziland, Tanzania and Zimbabwe [6,24,31,36,39,40,55,56,57,58]. Bark, leaf and root infusion is used against asthma in Malawi and Mozambique [13,55], candidiasis or oral candidiasis in Namibia and South Africa [27,59], chest complaints in South Africa and Swaziland [24,32,40,60], cough in Malawi, Mozambique, Namibia and South Africa [13,49,53,61], diarrhoea and stomach problems in Malawi, Mozambique, South Africa, Tanzania and Zimbabwe [13,20,37,38,39,52,55,62,63,64], induce labour and skin blisters in Mozambique [13] and blood pressure in Tanzania [51]. Leaf decoction of VI is used against abscesses and swellings in Malawi [55], dermatitis in Namibia [19,20] and toothache in South Africa [28,65]. Root decoction of VI is used as snake repellent or remedy for snake bites in Malawi and South Africa [29,55], remedy for infertility in Malawi and South Africa [55,66,67], epilepsy, measles and nervous system disorders in Malawi [55], inflammation of the umbilical cord and vaginal discharge in Zimbabwe [37,39], bewitchment, male virility in Tanzania and stomach ulcers in Tanzania [33,52] and pre-natal care in South Africa [68]. The fruit, root and seed decoction of VI is used against menstrual problems in South Africa, Tanzania and Zimbabwe [30,35,37,39,52,69]. Fruit, leaf and root decoction of VI is used against parasitic worms in Malawi, South Africa, Swaziland, Tanzania and Zimbabwe [28,31,34,39,55]. Leaf, root and seed decoction of VI is used against pneumonia in South Africa, Swaziland, Tanzania and Zimbabwe [6,28,31,35,39,52,56]. Bark decoction of VI is used as remedy for blood in stool in Swaziland [31,70] and syphilis in Tanzania [51] while leaf decoction is used against pleurisy and as protective wash against sorcery in Malawi [55]. In South Africa, roots of VI are mixed with those of *Bridelia micrantha* (Hochst.) Baill. and *Dichrostachys cinerea* (L.) Wight & Arn. and burnt with resultant smoke inhailed an anaesthetic [7] and roots are also mixed with those of *Helinus integrifolius* (Lam.) Kuntze as remedy for infertility [26].

## 4. Food Value

The fruits of VI are edible and highly sought-after by both humans and animals in east and southern Africa. In southern Africa, local people eat the fruits raw and dry them and save some for times in need and food scarcity [7,14]. Research by Chilimampunga [71] revealed that households in Mwanza district in Malawi use up to 40 kg of VI fruits per season with 53.5% of the households utilizing 6–15 kg of the fruits per season. The fruits are brown-orange when ripe and have an orange parenchyma tissue which is sweet, sour, somewhat bitter, slightly astringent and its taste has been compared to that of green apple (*Malus pumila* Miller), medlar (*Mespilus germanica* L.) and pineapple (*Ananas comosus* (L.) Merr.) [14]. Local communities in southern Africa prepare juice, jam and puddings by adding water and sugar or cook the fruits to make marmalade and the pulp can also be fermented to produce an alcoholic drink [7,14,28,65]. In South Africa and Swaziland, some households squeeze the fruit in water, discarding the skin and seeds, and use the juice for flavouring mealie meal porridge [65]. The edible fruits of VI are a good source of essential minerals such as calcium, copper, iron, magnesium, manganese, phosphorus, potassium and zinc (Table 3). When the fruits of VI are consumed, used as food additive or when other plant parts are used as herbal medicines, they may have an effect on human health.

Phytochemicals that have been identified from fruits, root bark, aerial shoots, leaves and roots of VI include alkaloids, anthraquinones, coumarins, glycosides, polyphenols, saponins, secoiridoids, steroids, tannins and terpenoids [32,54,72,73,74] and these phytochemicals are associated with several biological activities. Research by Chaves et al. [75] showed that alkaloids extracted from plants are characterized by anticholinesterase, antioxidant, anxiolytic, anti-inflammatory and antidepressant properties. The anthraquinones are characterized by antimicrobial and anti-inflammatory properties [76] while coumarins possess pharmacological activities like anti-HIV, antimicrobial, anti-inflammatory, anticancer, anti-TB, anticonvulsant, anti-depressant, anti-coagulant and antioxidant [77]. Research by Pandey and Rizvi [78] revealed that long term consumption of diets rich in plant polyphenols such as flavonoids, lignans, phenolic acids, quercetin and stilbenes offer protection against development of cancers, cardiovascular diseases, diabetes, osteoporosis and neuro-degenerative diseases. Saponins are bioactive compounds produced mainly by plants and they generally occur as glycosides of steroids or polycyclic triterpenes [79] and these compounds exhibit anticancer activities which include anti-proliferation, anti-metastasis, anti-angiogenesis, anti-multidrug resistance, and autophagy regulation actions [79,80]. Ono et al. [81] showed that secoiridoid isolated from *Verbena brasiliensis* Vell. exhibited antioxidative activities on 1,1-diphenyl-2-picrylhydrazyl (DPPH) radical than that of standard antioxidant, α-tocopherol. In Table 3, reference is also made to the recommended dietary allowance (RDA) representing the average daily intake of essential nutrients that are sufficient to meet the nutrient requirements of a health person. When nutritional composition values of VI are compared with RDA values, the species appear to be a good source of carbohydrates, copper, fibre, iron, magnesium, manganese, potassium, protein and Vitamin C (Table 3). Research by Raice et al. [15] showed that the fruit of VI has an attractive flavour and is rich in short fatty acids and ethyl and methyl esters. *Vangueria infausta* subsp. *infausta* is a typical nutraceutical and the phytochemicals present in the species may be responsible for a wide range of therapeutic effects against a number of diseases like abdominal pains [37,39,47], asthma [13,55], blood pressure [51], chest pains [24,32,40,60], cold [53,54], cough [13,49,53,54,55,61], diabetes [51], epilepsy [55], heartbeat problems [48,49], nervous system disorders [52], pneumonia [6,24,27,31,35,49,53] and stomach ulcers [33]. Gunther et al. [82] argued that people get more than basic nutritional needs from nutraceuticals like VI, as these plant species also have health-promoting and/or disease preventing properties leading to their use in treating and managing many disorders such as cancer, metabolic problems, cold and cough, depression, coronary heart disease, delayed gastrointestinal emptying, and many more conditions which need special care [83,84]. Similarly, Chivandi et al. [85] argued that edible fruits such as those derived from VI are important sources of macro- and micronutrients and health enhancing chemicals that have chemical, pharmacological and biological properties that mitigate some of the medical and biological effects of malnutrition.

## 5. Phytochemical Constituents and Nutritional Composition of *Vangueria infausta* Subsp. *infausta*

Several classes of phytochemicals such as biflavonoids, fatty acids, flavonoids, polyketide derivatives, iridoid lactones, pentacyclic triterpenoids and triterpenoid acids (Table 4). Barton et al. [92] identified triterpenoid acids such as tomentosolic acid and vanguerolic acid from the roots of VI (Table 4). Mbukwa et al. [93] isolated biflavonoid, 5,7,3′,5″,7″,4′′′-hexahydroxy (4′-*O*-3′′′)-biflavone, a polyketide derivative, methylcylohex-1-ene, flavonoids, (−)-epicatechin, epiafzelechin, dihydrokaempferol, quercetin, luteolin, dihydroquercetin-3′-*O*-glucoside, daidzein and genistein from aerial parts of VI. Abeer [94] isolated flavonoids luteolin-7-*O*-rutinoside, apigenin-7-*O*-rutinoside, luteolin-4-*O*-glucoside, quercetin-3-*O*-glucoside and quercetin from methanolic leaf extracts of VI. Raice et al. [17] identified fatty acids hexanoic acid and octanoic acid as well as fatty acid ethyl and methyl esters, ethyl hexanoate, ethyl octanoate, methyl hexanoate and methyl octanoate from fruits of VI. Bapela [95] isolated friedelin and morindolide from root extracts of VI (Table 4).

## 6. Pharmacological Activities of *Vangueria infausta* subsp. *infausta*

Based on *in vitro* and animal studies, a wide range of pharmacological activities of VI extracts and phytochemical compounds have been reported in literature and these include antibacterial [25,34,64,74,93,96,97], antimycobacterial [98,99], antifungal [34,74,93,100,101], anti-inflammatory [102], antileishmanial [103], antioxidant [93,94], antiplasmodial [58,72,73,95,104,105], antifeedant [106] and prostaglandin synthesis inhibitory [107].

### 6.1. Antibacterial Activity

De Boer et al. [34] evaluated the antibacterial activities of ethyl acetate, methanol and water leaf extracts of VI against *Erwinia amylovora*, *Pseudomonas syringae* and *Staphylococcus aureus* using microtiter plate assay. The extracts were active against the tested pathogens. Masoko [96] evaluated antibacterial activities of acetone, dichloromethane, hexane and methanolic leaf extracts of VI against *Enterococcus faecalis*, *Escherichia coli*, *Pseudomonas aeruginosa* and *Staphylococcus aureus* using serial dilution method with ampicillin as the positive control. The extracts showed activities against all pathogens except *Staphylococcus aureus*, with minimum inhibition concentration (MIC) values ranging from 0.02 to 1.25 mg/mL. The average total activity, a measure of potency for acetone, dichloromethane and methanolic extracts ranged from 135 mL/g to 531 mL/g [96]. Munodawafa et al. [74] evaluated antibacterial activities of leaf and root extracts of VI against *Escherichia coli*, *Pseudomonas aeruginosa*, *Staphylococcus aureus* and *Streptococcus* Group A using agar-well diffusion method with ampicillin (10 µg), amoxicillin (10 µg), gentamicin (10 µg) and tetramycin (10 µg) as positive controls. The extracts showed activities against *Escherichia coli*, *Pseudomonas aeruginosa* and *Streptococcus* Group A with the zone of inhibition ranging from 1.50 ± 0.58 mm to 3.25 ± 0.50 mm while the zone of inhibition exhibited by the controls ranged from 4.00 ± 0.0 mm to 13.00 ± 0.00 mm. The MIC values of extracts against *Escherichia coli*, *Pseudomonas aeruginosa*, *Staphylococcus aureus* and *Streptococcus* Group A ranged from 0.63 mg/mL to 10.0 mg/mL [74]. Shai et al. [97] evaluated antibacterial activities of acetone, dichloromethane and methanolic leaf extracts of VI against *Bacillus cereus*, *Bacillus stearothermophilus*, *Citrobacter fruendii*, *Enterobacter aerogenes*, *Enterococcus faecalis*, *Escherichia coli*, *Klebsiela oxytoca*, *Klebsiela pneumonia*, *Lactobacillus acidophilus*, *Micrococcus luteus*, *Proteus mirabilis*, *Proteus vulgaris*, *Pseudomonas aeruginosa*, *Pseudomonas fluorescens*, *Salmonella typhi*, *Serratia marcescens*, *Staphylococcus aureus*, *Staphylococcus epidermidis* and *Streptococcus pyogenes* using serial microplate dilution method with gentamicin as positive control. The extracts exhibited activities with MIC values ranging from 0.04 mg/mL to 2.5 mg/mL. The average total activity of the extracts ranged from 218 mL/g to 1531 mL/g [97]. Mthethwa et al. [25] evaluated antibacterial activities of VI root extracts against *Staphylococcus aureus* and *Staphylococcus epidermidis* using Kirby-Bauer disk diffusion and micro-dilution techniques with cloxacillin and DMSO as positive and negative controls, respectively. The extracts exhibited activities with zones of inhibition ranging from 20 mm to 24 mm. The MIC values ranged from 0.6 to 0.02 μg/mL [25]. Van Vuuren et al. [64] evaluated antibacterial activities of dichloromethane and methanol (1:1) and aqueous leaf extracts of VI against *Bacillus cereus*, *Enterococcus faecalis*, *Escherichia coli*, *Proteus vulgaris*, *Salmonella typhimurium*, *Shigella flexneri* and *Staphylococcus aureus* with ciprofloxacin as positive control. The extracts exhibited activities with MIC values ranging from 1.0 mg/mL to 8.0 mg/mL [64].

Mbukwa et al. [93] evaluated antibacterial activities of ethanol leaf, methanol stem bark extracts and compounds isolated from VI against *Bacillus subtilis*, *Escherichia coli* and *Staphylococcus aureus* using rapid agar overlay or immersion bioautography methods with chloramphenicol as positive control. The extracts, compounds epiafzelechin, (−)-epicatechin, genistein, luteolin and quercetin exhibited activities with MIC values ranging from 5.0 to 100 µg [93]. These documented antibacterial activities exhibited by both plant extracts and compounds isolated from the species support the traditional usage of the species as herbal medicine against bacterial infections such as diarrhoea and stomach problems [13,16,33,34,35,48,49,52,59,60,61], syphilis [48] and toothache [24,62].

### 6.2. Antimycobacterial Activity

Mmushi et al. [98] evaluated the antimycobacterial activities of acetone, dichloromethane, hexane and methanolic leaf extracts of VI against *Mycobacterium smegmatis* with rifampicin as the positive control. The extracts showed activities with MIC values ranging from 0.52 mg/mL to 1.25 mg/mL [98]. Similarly, Aro et al. [99] evaluated antimycobacterial activities of leaf extracts of VI against pathogenic *Mycobacterium tuberculosis* and non-pathogenic *Mycobacterium smegmatis*, *Mycobacterium aurum* and *Mycobacterium bovis* using a twofold serial microdilution assay. The MIC values of the extracts ranged from 0.23 mg/mL to 0.63 mg/ mL [99]. The results from antimycobacterial evaluations imply that the plant species may have potential anti-TB compounds, thus corroborating the use of the species against respiratory diseases such as chest complaints in South Africa and Swaziland [20,28,36,57] and cough in Malawi, Mozambique, Namibia and South Africa [13,46,50,51,52,58].

### 6.3. Antifungal Activity

De Boer et al. [34] evaluated antifungal activities of ethyl acetate, methanol, water leaf extracts of VI against *Aspergillus fumigatus*, *Candida albicans* and *Fusarium culmorum* using microtiter plate assay. The extracts were active against the tested pathogens. Munodawafa et al. [74] evaluated antifungal activities of leaf and root extracts of VI against *Candida albicans* and *Aspergillus niger* using agar-well diffusion method with amphotericin B (10 µg) as positive control. The extracts exhibited activities with the zone of inhibition ranging from 1.25 ± 0.50 mm to 2.00 ± 0.82 mm while the zone of inhibition exhibited by the control ranged from 6.35 ± 0.50 mm to 6.75 ± 0.58 mm. The MIC values of extracts ranged from 5.0 mg/mL to >10.0 mg/mL [74]. Mahlo et al. [100,101] evaluated antifungal activities of acetone, methanol, hexane and dichloromethane leaf extracts of VI against *Aspergillus niger*, *Aspergillus parasiticus*, *Colletotricum gloeosporioides*, *Fusarium oxysporum*, *Penicillium expansum*, *Penicillium janthinellum* and *Trichoderma harzianum* using micro-dilution assay with amphotericin B and 100% acetone as positive and negative controls, respectively. The extracts exhibited activities against all tested fungi species with MIC values ranging from 0.32 mg/mL to 2.50 mg/mL [100,101].

Mbukwa et al. [93] evaluated antifungal activities of ethanol leaf, methanol stem bark extracts and compounds isolated from VI against *Candida mycoderma* using rapid agar overlay or immersion bioautography methods with miconazole as control. The extracts, compounds 5,7,3′,5″,7″,4′′′-hexahydroxy (4′-*O*-3′′′)-biflavone, daidzein, dihydroquercetin-3′-*O*-glucoside, epiafzelechin, (−)-epicatechin, luteolin and quercetin exhibited activities with MIC values ranging from 0.1 µg to 50 µg [93]. These documented antifungal properties provide a scientific basis to the traditional uses of VI against fungal infections such as oral candidiasis in Namibia [56], candidiasis in South Africa [23] and other microbial infections.

### 6.4. Anti-Inflammatory Activity

Chowe [102] evaluated the *in vitro* anti-inflammatory activities of fruit ethanol extracts of VI using the denaturation of egg albumin protein and nitric oxide radical scavenging assay. The extracts showed activities with half maximal inhibitory concentration (IC_50_) value of 222.74 mg/L demonstrated by inhibition of egg albumin denaturation assay. The nitric oxide radical scavenging assay showed efficacy with IC_50_ value of 291.15 mg/L. The unsaponifiable fraction showed efficacy with IC_50_ value of 188.14 mg/L, while its flavonoids and standard drug indomethacin showed IC_50_ values of 663.44 mg/L and 119.34 g/L by egg albumin denaturation assay, respectively. The nitric oxide radical scavenging assay showed IC_50_ values of 616.60 mg/L and 1109.57 mg/L for the unsaponifiable fraction and flavonoids, respectively. The VI fruit had a comparable IC_50_ value with standard anti-inflammatory drug (indomethacin) and thus can be considered in the development of non-toxic anti-inflammatory remedies since they possess remarkable anti-inflammatory activity and as nutraceuticals which can be used as an additive in food [102]. These findings support the traditional use of VI in managing inflammatory ailments and diseases such as abdominal pains in Kenya and Zimbabwe [33,35,44], abscesses, pleurisy and swellings in Malawi [52], anaesthetic in South Africa [7], inflammation of umbilical cord in Zimbabwe [33,35], skin blisters in Mozambique [13] and stomach ulcers in Tanzania [29] and other problems that result in cell injury and death.

### 6.5. Antileishmanial Activity

Bapela et al. [103] evaluated antileishmanial activities of dichloromethane and methanol leaf extracts of VI against *Leishmania donovani*. The dichloromethane extracts displayed high inhibitory effects on the growth of amastigote forms of *Leishmania donovani* with IC_50_ values of 4.51 μg/mL [103]. Bapela et al. [55] demonstrated that most of the non-polar extracts of medicinal plants used in the treatment of malaria also possess significant antiplasmodial activities, and therefore, likely to have antileishmanicidal properties as both malaria and leishmaniasis are protozoal infections sharing several unique metabolic pathways. Therefore, findings of this research imply that VI extracts may have potential as antileishmanial agents.

### 6.6. Antioxidant Activity

Abeer [93] evaluated antioxidant activities of methanolic leaf extracts of VI using radical (DPPH), H_2_O_2_ scavenging activities and reducing power potential assay. The extracts revealed both free radical (DPPH), H_2_O_2_ scavenging activities and reducing power potential which was comparable to rutin, the standard [93]. Mbukwa et al. [92] evaluated antioxidant activities of ethanol leaf, methanol stem bark extracts and compounds isolated from VI using the DPPH assay with ascorbic acid as the control. The compounds 5,7,3′,5″,7″,4′′′-hexahydroxy-(4′-*O*-3′′′)-biflavone, luteolin, quercetin and dihydroquercetin-3′-*O*-glucoside exhibited half maximal effective concentration (EC_50_) values that were equal to or lower than 8.2 µg/mL exhibited by ascorbic acid, the standard. The extracts, dihydrokaempferol, epiafzelechin, (−)-epicatechin and genistein showed activities with EC_50_ values ranging from 15 µg/mL to 146 µg/mL, while EC_50_ value of daidzein was >146 µg/mL [92]. These antioxidant activities demonstrated by VI are probably due to the presence of short fatty acids [79], flavonoids and phenolics [28,51,69,70,71], since these phytochemical compounds have been isolated from the species.

### 6.7. Antiplasmodial Activity

Weneen et al. [104] evaluated antimalarial activities of dichloromethane, methanol and petroleum ether root extracts of VI against the multidrug resistant K1 strain of *Plasmodium falciparum*. The dichloromethane and methanol extracts demonstrated weak activity with IC_50_ value of 49.0 µg/mL and petroleum ether extract was inactive [104]. Nundkumar and Ojewole [72] evaluated antiplasmodial activities of the aqueous leaf extracts of VI using the parasite lactate dehydrogenase (pLDH) assay. The extract showed activity with IC_50_ value of 10–20 µg/mL [72]. Clarkson et al. [105] evaluated antiplasmodial activities of VI aqueous, dichloromethane, dichloromethane and methanol (1:1) fruit extracts against *Plasmodium falciparum* using the parasite lactate dehydrogenase (pLDH) assay. The dichloromethane and methanol (1:1) extract showed weak activity with IC_50_ value of 23.0 µg/mL [105].

Abosi et al. [73] evaluated in vivo antimalarial activities of VI root bark extracts against drug-sensitive *Plasmodium berghei* in mice using a standard inoculum of 1 × 107 infected erythrocytes and extracts of 500 mg/kg, 250 mg/kg or 125 mg/kg in a four-day suppression test and a Rane test of established infection. The extract showed antimalarial activity with a parasite suppression of 73.5% in early infection and a repository effect of 88.7% [73]. The same authors evaluated the antiplasmodial activities of the root extracts of VI by assessing the inhibition of *Plasmodium falciparum* growth by using the [3*H*]-hypoxanthine incorporation assay. The chloroform extract gave an IC_50_ value of 3.8 ± 1.5 µg/mL and 4.5 ± 2.3 µg/mL against D6 and W2 strains of *Plasmodium falciparum*, respectively, while butanol extract gave an IC_50_ value of 3.9 ± 0.3 µg/mL against the D6 strain, and chloroquine had an IC_50_ value of 0.016 µg/mL and 0.029 µg/mL against D6 and W2 strains, respectively [73]. Similarly, Bapela et al. [58] evaluated antiplasmodial activities of dichloromethane root extract of VI using the [3*H*]-hypoxanthine incorporation assay using chloroquine sensitive (NF54) strain of *Plasmodium falciparum* as the test organism. The extract showed significant activity with IC_50_ value of 1.84 μg/mL [58].

Bapela [95] evaluated antiplasmodial activities of compounds, friedelin, morindolide and an unknown compound X isolated from the roots of VI through the [3*H*]-hypoxanthine incorporation assay using chloroquine sensitive (NF54) strain of *Plasmodium falciparum* as the test organism. The compounds showed activities with friedelin, morindolide and compound X exhibiting IC_50_ values of 3.94 µg/mL, 18.5 µg/mL and 0.143 µg/mL, respectively [95]. These antiplasmodial activities demonstrated by VI support its use in the treatment and management of fever in Namibia and Tanzania [48,49,50,51] and malaria in Malawi, South Africa, Swaziland, Tanzania and Zimbabwe [6,20,27,32,33,36,52,53,54,55].

### 6.8. Antifeedant Activity 

Kaoneka and Mollel [106] evaluated the antifeedant activities of dichloromethane, methanol and petroleum ether extracts of the stem bark of VI against the anomalous emperor moth *Nudaurelia belina* using a modified leaf disc method. The dichloromethane and methanol extracts were found to be active against *Nudaurelia belina*. While these preliminary evaluations may serve as confirmation that VI has some bioactivities against ticks, a comprehensive method of tick control is required for the resource-constrained smallholder farmers based on ethnopharmacological properties of VI.

### 6.9. Prostaglandin Synthesis Inhibitory Activity

Lindsey et al. [107] evaluated aqueous and ethanol leaf extracts of VI for prostaglandin-synthesis inhibitors using the cyclooxygenase inhibitory bioassay. The aqueous and ethanol leaf extracts inhibited cyclooxygenase at 85% and 82%, respectively which were higher than 67% inhibition exhibited by the standard, indomethacin. The presence of prostaglandin-synthesis inhibitors in VI extracts support the usage of the plant species against menstrual problems in South Africa [55,57], Tanzania [31,49] and Zimbabwe [33,35].

### 6.10. Cytotoxicity Activity

Morobe et al. [46] evaluated cytotoxicity activities of methanolic and aqueous root extracts of VI against MAGI CC5+ cells using MTT (3-(dimethylthiozole-2-yl-2,5-diphenyltetrazolium bromide) assay. The extract exhibited half maximal cytotoxic concentration (CC_50_) value of 0.1 mg/mL [46]. Bapela et al. [58] evaluated cytotoxicity activities of dichloromethane and methanol (1:1) root extracts of VI against mammalian L-6 rat skeletal myoblast cells with podophyllotoxin as a control. The extract demonstrated IC_50_ value of 45.7 μg/mL and selectivity index value of 25 which was considered to be non-toxic to rat skeletal myoblast L6 cells [58]. Mthethwa et al. [25] evaluated cytotoxicity activities of VI root extracts using the MTT assay with berberine as positive control. The CC_50_ value of the extract was 100 μg/mL which was higher than 27 μg/mL exhibited by berberine, the control [25]. Aro et al. [99] evaluated cytotoxicity activities of leaf extracts of VI using MTT assay against C3A liver cells and Vero kidney cells. The half maximal lethal concentration (LC_50_) value against Vero kidney cells and C3A liver cells was 0.07 mg/mL and 0.76 mg/mL, respectively [99]. Bapela [95] evaluated cytotoxicity activities of compounds morindolide and an unknown compound X isolated from the roots of VI against mammalian L-6 rat skeletal myoblast cells with podophyllotoxin as a control. The compounds showed activities with morindolide and compound X exhibiting IC_50_ values of 61.5 µg/mL and 26.1 µg/mL, and the selectivity indices of morindolide and compound X were 3.32 and 182.5, respectively [95]. These preliminary cytotoxicity evaluations carried out so far [46,55,95,99,101] concluded that the species is characterized by low levels of cytotoxicity.

### 6.11. Toxicity Activity

Moshi et al. [108] evaluated toxicity of dichloromethane and ethanol leaf extracts of VI using the brine shrimp (*Artemia salina* L.) lethality test with cyclophosphamide as positive control. The ethanol extract exhibited LC_50_ values of 144.7 μg/mL which was considered to be non-toxic in comparison to the LC_50_ value of 16.3 μg/mL exhibited by cyclophosphamide, the positive control [108]. Munodawafa et al. [109] evaluated toxicity of leaf and root extracts of VI using the brine shrimp lethality test with *Nerium oleander* L. as positive control. The extracts exhibited LC_50_ values of 338 ± 23.4 μg/mL and 416 ± 28.3 μg/mL, respectively which were higher in comparison to the LC_50_ value of 141.7 μg/mL exhibited by *Nerium oleander*, the positive control [109].

## 7. Conclusions

*Vangueria infausta* subsp. *infausta* is widely used as food plant and herbal medicine in east and southern Africa. Documentation of the nutraceutical and ethnopharmacological properties of VI is crucial as this information forms the baseline data required for future research and development of food, health-promoting and pharmaceutical products. Findings from this study showed that there are still some research gaps in the phytochemistry, pharmacological and toxicological properties of the species. More rigorous research is required aimed at evaluating various plant parts used as food or herbal medicines, assessing their phytochemistry, pharmacological and toxicological properties. Preliminary phytochemical and pharmacological studies have provided supporting evidence for the therapeutic potential of VI in the management of malaria, bacterial, fungal and inflammatory problems. However, there is little or dearth of information on the majority of medical problems such as aphrodisiac, asthma, chest pains, cold, cough, dermatitis, diabetes, diarrhoea, epilepsy, fever, headache, heart problems, infertility, parasitic worms and antiviral effects. Therefore, in-depth research is required focusing on the molecular and cellular mechanisms of VI through identification of active chemical compounds, biological activities of these compounds and crude extracts of the species. Toxicological studies aimed at monitoring and confirming safety and efficacy of the compounds and crude extracts of VI should be followed by animals or in vivo studies as well as human clinical trials.

## Figures and Tables

**Table 1:** Vernacular names of *Vangueria infausta* subsp. *infausta* in the mainland tropical Africa.

Vernacular Name(s), Ethnic Group or Geographical Region in Brackets	Country	References
Wild meldlar (English), mmilo, monyonyana, mothwane, nzwigwa (Setswana)	Botswana	[9,10]
Mibiru (Kimîîru), orgomei (Maa)	Kenya	[11,12]
Mumzwiro (Chindau, Chitewe), African medlar, wild medlar (English), maphilwa, n’pfilwa (Ronga)	Mozambique	[13,14,15,16,17]
Omundjenja (Herero), oshimbu (Oshiwambo)	Namibia	[6,18,19,20]
Grootmispel, mispel, wildemispel (Afrikaans), velvet wild medlar, wild medlar (English), umbizo, umviyo (Ndebele), mmilo (Northern Sotho), mpfilwa (Shangaan, Tsonga), xinyathelo (Tsonga), amantulwane, umntuli, umntulwa, umvile (Swati), mmilo, mothwanyê (Tswana), mavelo, muzwilo, muzwilu (Venda), umvilo, umviyo (Xhosa), idulumuthwa, inkhabayomtwana, isantulu-tshwana, umfilwa, umgana, umsunuwengane, umtulwa, umviki, umviyo (Zulu)	South Africa	[6,7,8,21,22,23,24,25,26,27,28,29,30]
Wild medlar (English), imadnulu, imandulu, infahlo, infaylo, infaylo, infayo, limandvulo, mantulwa, mavelo, santudlevane, santulwan, santulwana, santulwane, umfilwa, umgana, umntudlwana, umntuli, umntulu, umntulwa, umvigo, umvile, umviyo (Siswati)	Swaziland	[31,32]
Amabungo, mtugunda (Kagera, Lindi), mviru (Kiluguru), mdaria (Kipare), msada, mvilu (Zigua)	Tanzania	[33,34,35,36]
False medlar, velvet wild medlar (English), umthofu, umviyo (Ndebele), mudzvirungombe, munjiro, munzviro, munzirwa, munzvirwa, mutsviru (Shona)	Zimbabwe	[2,20,37,38,39]

**Table 2:** Ethnomedicinal uses of *Vangueria infausta* subsp. *infausta* in tropical Africa.

Use	Plant Parts Used	Country Practiced	References
Abdominal pains	Root infusion taken orally	Kenya, Zimbabwe	[37,39,47]
Abscesses	After abscess is drained, leaf infusion is applied topically	Malawi	[55]
Anaesthetic	Smoke from burnt roots inhailed mixed with roots of *Bridelia micrantha* (Hochst.) Baill. and *Dichrostachys cinerea* (L.) Wight & Arn.	South Africa	[7]
Aphrodisiac	Leaf, root decoction taken orally	South Africa, Tanzania	[6,24,33,40,50]
Asthma	Bark, leaf, root infusion taken orally	Malawi, Mozambique	[13,55]
Bewitchment	Root decoction taken orally	Tanzania	[51]
Blood pressure	Bark, leaf, root decoction taken orally	Tanzania	[51]
Bloody stool	Bark infusion taken orally	Swaziland	[31,70]
Chest pain	Bark, leaf, root decoction taken orally	South Africa, Swaziland	[24,32,40,60]
Cold	Leaf, root, shoots infusion taken orally	Namibia	[53,54]
Cough	Bark, leaf, root infusion taken orally	Malawi, Mozambique, Namibia, South Africa	[13,49,53,54,55,61]
Dermatitis	Leaf infusion applied topically	Namibia	[19,20]
Diabetes	Leaf, root decoction taken orally	Tanzania	[51]
Diarrhoea and stomach problems	Bark, leaf, root decoction taken orally	Malawi, Mozambique, South Africa, Tanzania, Zimbabwe	[13,20,37,38,39,51,52,55,62,64]
Epilepsy	Root decoction taken orally	Malawi	[55]
Fever	Leaf, root, shoots decoction taken orally	Namibia, Tanzania	[51,52,53,54]
Headache	Leaf, root, shoot decoction taken orally	Namibia	[53,54]
Hernia	Leaf, root decoction taken orally	Tanzania	[52]
Induce labour	Bark, leaf, root decoction taken orally	Mozambique	[13]
Infertility	Root decoction taken orally mixed with roots of *Helinus integrifolius* (Lam.) Kuntze	South Africa	[26]
Infertility	Bark, root decoction taken orally	Malawi, South Africa, Tanzania	[51,55,66,67]
Inflammation of umbilical cord	Root decoction taken orally	Zimbabwe	[37,39]
Malaria	Leaf, root decoction taken orally	Malawi, South Africa, Swaziland, Tanzania, Zimbabwe	[6,24,31,36,39,40,55,56,57,58]
Male virility	Root decoction taken orally	Tanzania	[52]
Measles	Root decoction applied topically	Malawi	[55]
Menstrual problems	Fruit, root, seed decoction taken orally	South Africa, Tanzania, Zimbabwe	[30,35,37,39,52,69]
Nervous system disorders	Root decoction taken orally	Malawi	[55]
Oral candidiasis or candidiasis	Bark, leaf, root decoction taken orally	Namibia, South Africa	[27,59]
Parasitic worms	Fruit, leaf, root decoction taken orally	Malawi, South Africa, Swaziland, Tanzania, Zimbabwe	[28,31,35,39,51,55]
Pleurisy	Leaf decoction taken orally	Malawi	[55]
Pneumonia	Leaf, root, seed decoction taken orally	South Africa, Swaziland, Tanzania, Zimbabwe	[6,28,31,35,39,52,56]
Pre-natal care	Root decoction taken orally	South Africa	[68]
Protective wash against sorcery	Leaf decoction taken orally	Malawi	[55]
Purgative	Leaf, root decoction taken orally	South Africa, Swaziland	[28,31]
Skin blisters	Bark, leaf, root decoction applied topically	Mozambique	[13]
Slow down heartbeat	Root decoction taken orally	Botswana, South Africa	[48,49]
Snake repellent or remedy for snake bites	Root decoction sprayed around homestead or applied topically on the place of the bite	Malawi, South Africa	[29,55]
Stomach ulcers	Root decoction decoction taken orally	Tanzania	[33]
Swellings	Leaf decoction applied topically	Malawi	[55]
Syphilis	Bark decoction taken orally	Tanzania	[51]
Toothache	Leaf decoction applied topically	South Africa	[28]
Virginal discharge	Root decoction applied topically	Zimbabwe	[37,39]

**Table 3:** Nutritional composition of fruits and other plant parts of *Vangueria infausta* subsp. *infausta*.

Caloric and Nutritional Composition	Values	Plant Parts	Recommended Dietary Allowance (RDA)	Reference
Acid detergent fibre (ADF) (%)	19.0–39.5	Fruits, seeds	-	[86,87]
Ash (g/100g dry matter)	2.6–5.5	Fruits, pulp, seeds	-	[16,86,87,88,89]
Ca (mg/100g)	0.20–186.33	Fruits, seeds	1000–1300	[16,86,87,88,89,90]
Carbohydrate (%)	77.07–78.10	Fruits	45–65	[88,89]
Crude fat (g/100g dry matter)	0.5–7.0	Fruits, pulp, seeds	300	[16,86,88]
Crude fibre (g/100g dry matter)	10.20–10.29	Fruits	25–38	[88,89]
Crude protein (g/100g dry matter)	3.01–21.30	Fruits, pulp, seeds	34	[16,86,88,89,90]
Cu (mg/100g)	5.91–10.1	Fruits, seeds	1–2	[86]
Dry matter (%)	19.30–97.10	Fruits, pulp, seeds	-	[16,86,87,88,89,90]
Energy value (Kj/100g	1445	Fruits	-	[88]
Fe (mg/100g)	0.09–21.60	Fruits, seeds	8–15	[86,87,88,89,90]
Fructose (g/100g)	1.4	Pulp	130	[16]
Glucose (g/100g)	1.4	Pulp	130	[16]
K (mg/100g)	1.80–1683.00	Fruits	4700	[87,88,89,90]
Mg (mg/100g)	0.06–99.00	Fruits, seeds	310–320	[86,87,88,89]
Mn (mg/100g)	2.91–47.40	Fruits, seeds	5	[86,89,90]
Moisture (%)	4.16–80.70	Fruits	-	[89,90]
N (mg/100g)	0.90	Fruits	-	[90]
Na (mg/100g)	13.70–160.81	Fruits	2300	[87,88,89]
Neutral detergent fibre (NDF) (%)	12.40–39.40	Fruits, seeds	-	[86,87]
P (mg/100g)	3.50–86.86	Fruits, seeds	1250	[16,86,87,88,89,90]
Phenolic content (gallic acid equivalent mg/mL)	444. 07	Aerial shoots	-	[54]
Sucrose (g/100g)	2.7	Pulp	130	[16]
Tannin (mg/mL gallic acid equivalent)	4.08	Leaf	-	[91]
Vitamin C (mg/100g)	11.50–67.70	Fruits	46	[87,89]
Zn (mg/100 g)	0.02–0.16	Fruits, seeds	8–11	[86,87]

**Table 4:** Chemical compounds isolated and characterized from *Vangueria infausta* subsp. *infausta*.

Compound	Plant Part	Isolation and Identification Method	References
**Biflavonoid**			
5,7,3′,5″,7″,4′′′-hexahydroxy (4′-*O*-3′′′)-biflavone	Leaves, stem bark	ESI-MS, IR, NMR, UV	[93]
**Fatty acid**			
Hexanoic acid (5600 µg/g of dry matter)	Fruits	GC-MS	[17]
Octanoic acid (240 µg/g of dry matter)	Fruits	GC-MS	[17]
**Fatty acid ethyl and methyl esters**			
Ethyl hexanoate (44 µg/g of dry matter)	Fruits	GC-MS	[17]
Ethyl octanoate (13 µg/g of dry matter)	Fruits	GC-MS	[17]
Methyl hexanoate (15 µg/g of dry matter)	Fruits	GC-MS	[17]
Methyl octanoate (12 µg/g of dry matter)	Fruits	GC-MS	[17]
**Flavonoids**			
Apigenin-7-*O*-rutinoside (22 mg/g of dry matter)	Leaves	NMR, UV	[94]
Daidzein	Leaves, stem bark	ESI-MS, IR, NMR, UV	[93]
Dihydrokaempferol	Leaves, stem bark	ESI-MS, IR, NMR, UV	[93]
Dihydroquercetin-3′-*O*-glucoside	Leaves, stem bark	ESI-MS, IR, NMR, UV	[93]
Epiafzelechin	Leaves, stem bark	ESI-MS, IR, NMR, UV	[93]
(−)-epicatechin	Leaves, stem bark	ESI-MS, IR, NMR, UV	[93]
Genistein	Leaves, stem bark	ESI-MS, IR, NMR, UV	[93]
Luteolin	Leaves, stem bark	ESI-MS, IR, NMR, UV	[93]
Luteolin-7-*O*-rutinoside (25 mg/g of dry matter)	Leaves	NMR, UV	[94]
Luteolin-4-*O*-glucoside (14 mg/g of dry matter)	Leaves	NMR, UV	[94]
Quercetin (10 mg/g of dry matter)	Leaves, stem bark	ESI-MS, IR, NMR, UV	[93,94]
Quercetin-3-*O*-glucoside (18 mg/g of dry matter)	Leaves	NMR, UV	[94]
**Polyketide derivative**			
Methylcylohex-1-ene	Leaves, stem bark	ESI-MS, IR, NMR, UV	[93]
**Iridoid lactone**			
Morindolide (4 mg/g of dry matter)	Roots	NMR, TLC	[95]
**Pentacyclic triterpenoid**			
Friedelin (7 mg/g of dry matter)	Roots	NMR, TLC	[95]
**Triterpenoid acids**			
Tomentosolic acid	Roots	TLC	[91]
Vanguerolic acid	Roots	TLC	[91]
